# Ki67表达及*EGFR*突变对Ⅰ期肺腺癌患者术后复发转移风险的相关分析

**DOI:** 10.3779/j.issn.1009-3419.2022.101.55

**Published:** 2022-12-20

**Authors:** 丽莉 许, 人花 郭

**Affiliations:** 210029 南京，南京医科大学第一附属医院肿瘤科 Department of Oncology, First Affiliated Hospital of Nanjing Medical University, Nanjing 210029, China

**Keywords:** Ki67, 表皮生长因子受体, 肺肿瘤, 预后, Ki67, Epidermal growth factor receptor, Lung neoplasms, Prognosis

## Abstract

**背景与目的:**

尽管多数Ⅰ期非小细胞肺癌（non-small cell lung cancer, NSCLC）术后患者的预后良好，但仍有部分可能早期复发转移，目前临床尚无标准方法筛选这部分人群。本研究探讨了Ⅰ期肺腺癌术后患者Ki67的表达及表皮生长因子受体（epidermal growth factor receptor, *EGFR*）突变状态与其复发风险的关系。

**方法:**

对118例Ⅰ期肺腺癌患者进行回顾性调查，将患者术后标本用免疫组化法（immunohistochemistry, IHC）检测Ki67的表达水平，扩增阻滞突变系统-链式聚合酶反应法（amplification refractory mutation system polymerase chain reaction, ARMS-PCR）或直接测序法检测*EGFR*基因突变状态，并收集一般临床资料。采用*Kaplan-Meier*法、*Log-rank*检验、*Cox*比例风险回归模型进行预后统计分析。

**结果:**

118例患者中，Ki67高表达率为43.22%（51/118），与性别、是否吸烟、手术方式、分化程度、术后分期相关（*P* < 0.05）；*EGFR*突变率61.02%（72/118），其中*EGFR*外显子19缺失突变率19.49%（23/118），*EGFR*外显子21 L858R突变率41.53%（49/118），女性、非吸烟者更容易出现*EGFR*突变（*P* < 0.05）。Ki67表达与*EGFR*突变状态无显著相关（*χ*^2^=1.412, *P*=0.235）。生存分析提示Ki67高表达与Ⅰ期肺腺癌术后无病生存期（disease-free survival, DFS）及总生存期（overall survival, OS）呈负相关（*P* < 0.05）；*EGFR*突变状态与Ⅰ期肺腺癌术后DFS、OS无显著相关（*P* > 0.05），而亚组分析显示，相较于*EGFR*外显子21 L858R突变组，*EGFR*外显子19缺失组的5年DFS显著降低，差异有统计学意义（*P*=0.031），但在OS上并无统计学差异（*P*=0.308）。多因素分析发现Ki67表达（*P*=0.001）对Ⅰ期肺腺癌术后DFS影响具有统计学意义，Ki67表达（*P*=0.03）、性别（*P*=0.015）对Ⅰ期肺腺癌术后OS影响具有统计学意义。

**结论:**

Ki67表达是Ⅰ期肺腺癌术后复发及OS的独立影响因素，与*EGFR*突变无明显相关；本研究中，*EGFR*突变状态与Ⅰ期肺腺癌预后未见显著相关，但不同*EGFR*突变类型预后不同，相较于*EGFR*外显子21 L858R突变，*EGFR*外显子19缺失患者的术后复发风险更高。

## Abstract (en)

**Background and objective:**

The prognosis of stage I non-small cell lung cancer (NSCLC) is generally good. However, some of the stage I NSCLC patients still may have early recurrence and metastasis, and there is no standard method to screen this part of the population. The aim of this study is to investigate the relationship between Ki67 expression as well as epidermal growth factor receptor (*EGFR*) mutation and the risk of recurrence in postoperative patients with stage I lung adenocarcinoma.

**Methods:**

We retrospectively enrolled 118 postoperative patients with stage I lung adenocarcinoma. *EGFR* mutation was tested using amplification refractory mutation system polymerase chain reaction (ARMS-PCR), and Ki67 level was detected by immunohistochemistry (IHC), followed by the collection of the patients' clinical characteristics. *Kaplan-Meier* method, *Log-rank* test, and *Cox* proportional hazards regression model were used for the prognostic statistical analysis.

**Results:**

Among the 118 patients, the rate of high Ki67 expression was 43.22% (51/118), which is related to gender, smoking status, surgical method, differentiation degree, and postoperative stage (*P* < 0.05). Meanwhile, *EGFR* mutation rate was 61.02% (72/118), of which *EGFR* exon 19 deletion mutation rate was 19.49% (23/118), and the *EGFR* exon 21 L858R mutation rate was 41.53% (49/118). However, Ki67 expression was not associated with *EGFR* mutation status (*χ*^2^=1.412, *P*=0.235). Survival analysis showed that high Ki67 expression was inversely associated with disease-free survival (DFS) and overall survival (OS) in stage I lung adenocarcinoma (*P* < 0.05), but *EGFR* mutation status was not significantly associated with DFS and OS (*P* > 0.05). In the subgroup analysis, the DFS of the *EGFR* exon 19 deletion group was significantly decreased compared with the *EGFR* exon 21 L858R mutation group (*P*=0.031), but there was no significant difference in OS (*P*=0.308). Multivariate analysis showed that there was statistical significance between Ki67 expression (*P*=0.001) and DFS in stage I lung adenocarcinoma; Ki67 expression (*P*=0.03) and gender (*P*=0.015) were associated with OS in stage I lung adenocarcinoma.

**Conclusion:**

Ki67 expression is an independent influencing factor for postoperative recurrence and OS of stage I lung adenocarcinoma and it is not significantly associated with *EGFR* mutation. There is no significant difference between *EGFR* mutation status and the prognostis of stage I lung adenocarcinoma. However, the prognosis differed in *EGFR* mutation types; the patients with *EGFR* exon 19 deletion are at higher risk of recurrence than *EGFR* exon 21 L858R mutation.

肺癌是全球最常见的威胁人类健康的恶性肿瘤之一，其发病率及死亡率均逐年增长，其中非小细胞肺癌（non-small cell lung cancer, NSCLC）约占原发性肺癌的85%，腺癌作为NSCLC最常见的病理类型，约占50%^[1]^。然而相同病理类型及分期的肺癌，无论经手术或内科治疗，其疗效和预后也存在较大差异。

在临床实践中发现，尽管通过手术完全切除，仍有很多Ⅰ期NSCLC术后5年内复发，部分不足1年即复发，约1/3的患者死于肺癌复发转移。据报道^[2]^，Ⅰ期肺癌患者平均5年生存率约78.6%，其中IA1期约92%，IA2期约83%，IA期约77%，IB期约68%。对于Ⅰ期NSCLC患者，一线治疗方案是根治性手术，目前无临床研究支持IA期患者行术后辅助化疗；IB期患者术后辅助化疗仍存争议，而基于ADAURA研究^[3]^，2021年美国国家综合癌症网络（National Comprehensive Cancer Network, NCCN）指南首次推荐对于表皮生长因子受体（epidermal growth factor receptor, *EGFR*）突变的IB期NSCLC患者术后予奥希替尼作为辅助靶向治疗。但目前尚无标准方法预测Ⅰ期NSCLC患者术后的复发风险。

Ki67是一种增殖细胞相关的核抗原，在多种恶性肿瘤中高表达，可反映肿瘤细胞的增殖率、恶性程度及侵袭力，与肿瘤的发生、发展及预后密切相关^[4]^，已被广泛应用于评估多种肿瘤分裂细胞比例与肿瘤分级，如乳腺癌^[5]^、神经内分泌肿瘤^[6-8]^等，但Ki67对NSCLC预后预测价值仍存在争议。

EGFR是原癌基因*CerbB-1*的表达产物，参与调节恶性肿瘤细胞的存活、分化、增殖、侵袭、转移及新血管生成等^[9]^。先前的基因组相关研究^[10, 11]^，早已证实EGFR在肺癌中普遍存在变异，在指导肺癌的治疗和判断患者预后中发挥了重要作用。但既往多数研究模糊了EGFR酪氨酸激酶抑制剂（tyrosine kinase inhibitors, TKIs）药物后对肺癌预后的影响，*EGFR*突变状态与早期肺腺癌患者术后复发风险的纯预后作用尚存在争议。

基于此，本研究回顾性分析了118例早期肺腺癌患者的临床数据资料，旨在研究Ki67的表达及*EGFR*突变状态与早期NSCLC术后复发及总生存的相关性，以期筛选高危人群，给予合适的干预，从而提高这部分人群的生存率。

## 1 资料与方法

### 1.1 临床资料

收集2012年1月-2014年12月在南京医科大学第一附属医院行手术治疗，且按美国癌症联合会（America Joint Committee for Cancer, AJCC）第8版肿瘤原发灶-淋巴结-转移（tumor-node-metastasis, TNM）分期标准重新分期为Ⅰ期的肺腺癌患者233例，取术后肿瘤组织样本检测Ki67表达，并从本院分子检测资料库中查找已行EGFR检测的病例。所有患者术前均行常规颅脑计算机断层扫描（computed tomography, CT）、胸腹部CT和骨放射性核素断层扫描（emission computed tomography, ECT）等检查，以排除转移。如CT等显示存在问题，则另行磁共振成像（magnetic resonance imaging, MRI）、正电子发射型计算机断层显像（positron emission computed tomography, PET）/CT或活检等。通过排除，最终入选118例。排除标准：①术前行放化疗、靶向治疗、免疫治疗等新辅助治疗；②手术未行淋巴结清扫；③多中心灶，仅单病灶切除；④合并*EGFR*少见突变或*EGFR*突变类型未知；⑤合并其他恶性肿瘤；⑥资料不完善者；⑦失随访者或因其他原因死亡者。本研究得到了南京医科大学第一附属医院伦理委员会的批准。

### 1.2 研究方法

#### 1.2.1 免疫组化

肿瘤组织予10%福尔马林溶液固定，石蜡包埋，制作厚度为4 μm的组织切片。采用MaxVision^TM^即用型快速免疫组化一步法检测各肿瘤组织中Ki67表达情况，于一抗前进行抗原热修复20 min，室温下冷却，DAB显色后行苏木精对照染色。以PBS代替一抗作为阴性对照。显微镜下观察，组织切片上棕褐色为阳性细胞核，蓝紫色为阴性细胞核，使用软件计数阳性细胞比例。试剂盒、抗体等均购于福州迈新生物技术开发公司。

#### 1.2.2 *EGFR*基因突变检测

由于为回顾性研究，本院2012年-2015年主要应用扩增阻滞突变系统-链式聚合酶反应法（amplification refractory mutation system polymerase chain reaction, ARMS-PCR）检测*EGFR*基因突变，前期部分予直接测序法。因检测年份较早，受检测方法局限，将其中*EGFR*外显子19缺失或*EGFR*外显子21 L858R突变为*EGFR*突变组，剔除少见*EGFR*突变，无相关突变的为EGFR野生型组。

患者术后病理资料、Ki67表达及*EGFR*基因突变状态结果均由两名或两名以上病理医师确认。

#### 1.2.3 随访

通过医疗记录或电话术后随访患者，随访时间自手术之日起至末次随访或死亡时间，时间14个月-122个月，随访截止时间：2022年6月30日。随访方法：术后连续2年每3个月复查一次，第3-5年每半年复查一次，以后每年复查一次，检查内容包括胸片、CT、B超、MRI、骨ECT、PET/CT、肿瘤标志物等。随访主要终点为无病生存期（disease-free survival, DFS），次要终点为总生存期（overall survival, OS）。DFS：从手术当天开始至疾病复发或因任何原因死亡的时间。OS：从手术当天开始至因任何原因死亡时间。考虑DFS、OS作为终点较难记录，随访要求高，需及时发现肿瘤复发，肿瘤患者死亡原因难以确定，因此本次随访过程失访或死于其他疾病患者不考虑进一步评估。由于为回顾性研究，Ⅰ期NSCLC术后辅助治疗证据不足，行术后辅助治疗的患者半数以上为IB期患者，部分为自我意愿。对于复发的患者，后续治疗包括手术切除、放疗、化疗、靶向治疗及免疫治疗等。

### 1.3 统计学方法

采用GraphPad Prism 7.0或SPSS 26.0统计学软件进行数据分析，使用*Pearson*卡方检验和*Fisher*精确检验分析Ki67表达、*EGFR*基因突变状态与临床资料参数之间的差异；采用受试者工作特征（receiver operating characteristic, ROC）曲线分析连续变量Ki67，并计算其Cut-off值；DFS、OS用*Kaplan-Meier*生存曲线描述，生存率差异用*Log-rank*检验；*Cox*比例风险回归模型用于评估危险因素；*P* < 0.05为差异具有统计学意义。

## 2 结果

### 2.1 临床资料

118例Ⅰ期肺腺癌术后病例中，男性52例，女性66例；年龄38岁-83岁，中位年龄62岁；术后行辅助治疗24例（包括22例化疗，2例靶向治疗），其中IB期14例，IA2期、IA3期各5例。术后患者临床资料见表 1。截止至随访日期，Ⅰ期肺腺癌患者的中位DFS及OS未达到。随访到29例复发转移，占总人数的24.58%，其中5年内复发24例（20.34%），大于5年复发5例（4.24%）；死亡13例，占总人数的11.02%，其中5年内死亡7例（5.93%），大于5年死亡6例（5.08%）。

**表 1:** Ki67表达和*EGFR*基因突变状态与临床特征的关系 Ki67 expression and *EGFR* mutation status in relation to clinical characteristics

Characteristics	*n* (%)	EGFR [*n* (%)]		*P*		Ki67 [*n* (%)]		*P*
		Mutant type (*n*=72)	Wild type (*n*=46)			High expression (*n*=51)	Low expression (*n*=67)	
Age (year)				0.323				0.187
< 60	45 (38.14)	30 (41.67)	15 (32.61)			16 (31.37)	29 (43.28)	
≥60	73 (61.86)	42 (58.33)	31 (67.39)			35 (68.63)	38 (56.52)	
Gender				0.003				0.015
Female	66 (55.93)	48 (66.67)	18 (39.13)			22 (43.14)	44 (65.67)	
Male	52 (44.07)	24 (33.33)	28 (60.87)			29 (56.86)	23 (34.33)	
Smoking status				0.003				0.018
Never	89 (75.42)	61 (84.72)	28 (60.87)			33 (64.71)	56 (83.58)	
Current or ever	29 (24.58)	11 (15.28)	18 (39.13)			18 (35.29)	11 (16.42)	
Surgical method				0.731				0.029
Lobectomy	93 (78.81)	56 (77.78)	37 (80.43)			45 (88.24)	48 (71.64)	
Segmentectomy	25 (21.19)	16 (22.22)	9 (19.57)			6 (11.76)	19 (28.36)	
Differentiation degree				0.093				0.001
High	55 (46.61)	38 (52.78)	17 (36.96)			15 (29.41)	40 (59.70)	
Medium and low	63 (53.39)	34 (47.22)	29 (63.04)			36 (70.59)	27 (40.30)	
Stage				0.557				0.032
IA1	12 (10.17)	8 (11.11)	4 (8.70)			1 (1.96)	11 (16.42)	
IA2	47 (39.83)	31 (43.06)	16 (34.78)			20 (39.22)	27 (40.30)	
IA3	28 (23.73)	14 (19.44)	14 (30.43)			12 (23.53)	16 (23.88)	
IB	31 (26.27)	19 (26.39)	12 (26.09)			18 (35.29)	13 (19.40)	
Pleural invasion				0.970				0.089
Yes	28 (23.73)	17 (23.61)	11 (23.9)			16 (31.37)	12 (17.91)	
No	90 (76.27)	55 (76.39)	35 (76.1)			35 (68.63)	55 (82.09)	
Tumor diameter (cm)				0.573				0.060
≤1	10 (8.47)	7 (9.72)	3 (6.52)			1 (1.96)	9 (13.43)	
1-2	64 (54.24)	40 (55.56)	24 (52.17)			28 (54.90)	36 (53.73)	
2-3	39 (33.05)	21 (29.17)	18 (39.13)			18 (35.29)	21 (31.34)	
> 3	5 (4.24)	4 (5.56)	1 (2.17)			4 (7.84)	1 (1.49)	
Postoperative treatment				0.525				0.772
Yes	24 (20.34)	16 (22.22)	8 (17.39)			11 (21.57)	13 (19.40)	
No	94 (79.66)	56 (77.78)	38 (82.61)			40 (78.43)	54 (80.60)	
EGFR: epidermal growth factor receptor.								

### 2.2 Ki67表达情况

由于目前在NSCLC中没有Ki67 Cut-off值的共识，通过ROC曲线分析Ki67不同表达量，判断Ⅰ期肺腺癌患者术后复发及总生存预后的灵敏度及特异度，计算约登指数，取最大值所对应的点为最佳临界值。我们获得了118例Ⅰ期肺腺癌患者的Ki67 Cut-off值。Ki67（5年DFS）的ROC曲线下面积（area under the curve, AUC）为0.788，Cut-off值为19.4%，灵敏度及特异度分别为72.00%、77.42%（*P* < 0.001）（图 1A）；而Ki67（OS）的AUC为0.810，Cut-off值为20.0%，灵敏度及特异度分别为76.92%、80.00%（*P* < 0.001）（图 1B）。两者均有统计学意义，数值相近，选择19.4%作为Ki67 Cut-off值。118例中有51例（43.22%）为高表达，67例（56.78%）为低表达。

**图 1:**
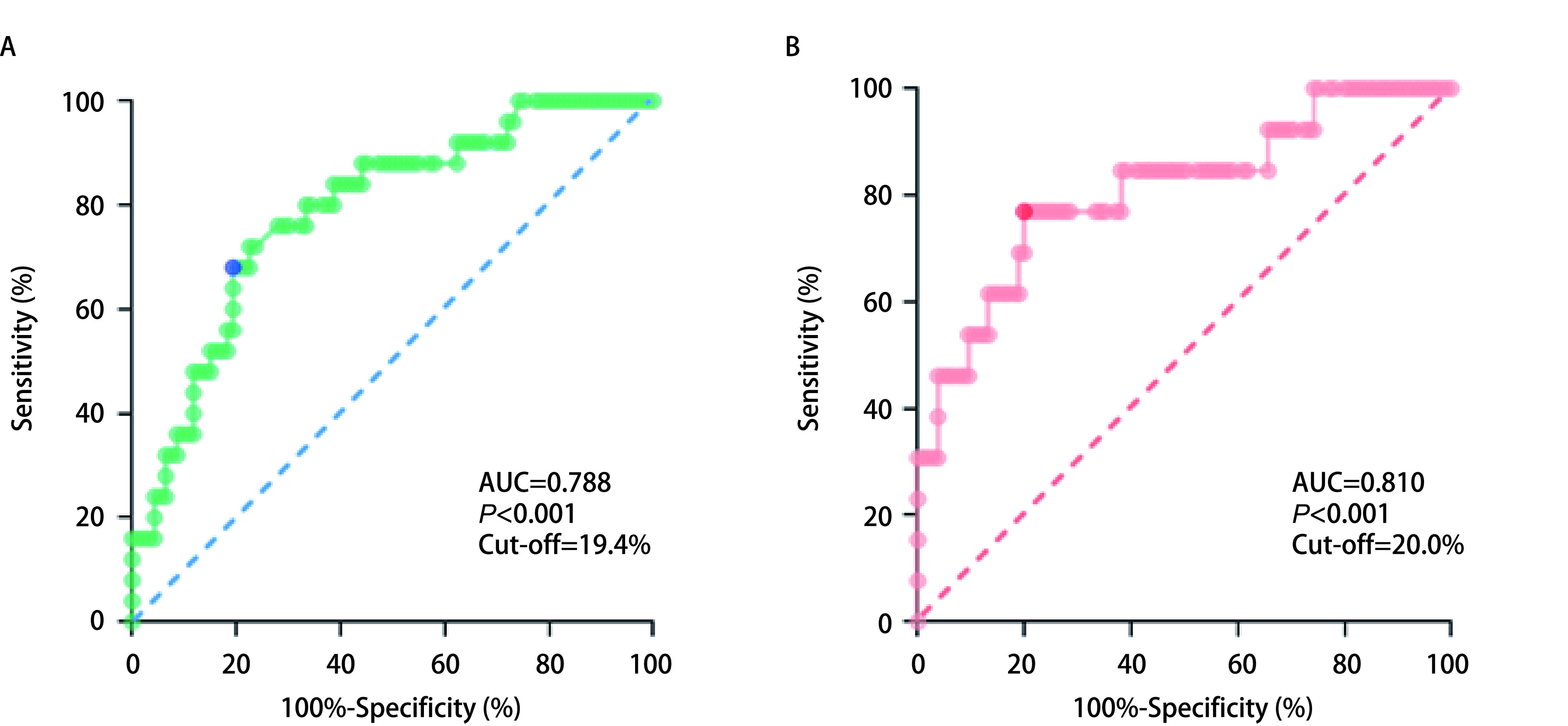
118例Ⅰ期肺腺癌患者Ki67的ROC曲线。A：Ki67 5年DFS的ROC曲线；B：Ki67 OS的ROC曲线。 ROC curve of Ki67 in 118 patients with stage I lung adenocarcinoma. A: The ROC curve of Ki67 in 5-year DFS; B: The ROC Curve of Ki67 in OS. ROC: receiver operating characteristic curve; OS: overall survival; DFS: disease-free survival; AUC: area under the curve.

### 2.3 Ki67表达与临床特征之间的关系

Ki67高表达与男性（*P*=0.015）、吸烟（*P*=0.018）、肺叶切除（*P*=0.029）、较差的分化（*P*=0.001）、较高的病理分期（*P*=0.032）等临床参数显著相关；与年龄、胸膜侵犯、肿瘤直径等无关（*P* > 0.05）（表 1）。

### 2.4 *EGFR*基因突变状态

排除*EGFR*少见突变后，118例中有46例为EGFR野生型，72例有*EGFR*突变，总检出突变率为61.02%，其中*EGFR*外显子19缺失23例，占19.49%；*EGFR*外显子21 L858R突变49例，占41.53%。

### 2.5 *EGFR*基因突变状态与临床特征之间的关系

*EGFR*基因突变检测中女性突变率高于男性，不吸烟患者突变率高于吸烟者，差异有统计学意义（*P*=0.003）；*EGFR*基因突变与Ⅰ期肺腺癌年龄、肿瘤直径、手术方式、术后分期、分化程度、有无侵犯胸膜等无关（表 1）。

### 2.6 Ki67表达与EGFR基因突变状态的关系

在118例病例中，Ki67高表达、EGFR突变型28例（23.73%），Ki67高表达、EGFR野生型者23例（19.49%），Ki67低表达、EGFR突变型44例（37.29%），Ki67低表达、EGFR野生型23例（19.49%）。Ki67表达与EGFR基因突变状态之间无明显关系（*P*=0.235），同样Ki67表达与EGFR不同基因突变类型之间亦未发现相关性（*P*=0.584）（表 2）。

**表 2:** Ki67表达与*EGFR*基因突变的关系 The relation between Ki67 expression and *EGFR* mutation status

Ki67	*EGFR* [*n* (%)]					*EGFR* mutant type [*n* (%)]			
	Mutant type	Wild type	*χ* ^2^	*P*		19del+	L858R+	*χ* ^2^	*P*
High expression	28 (23.73)	23 (19.49)	1.412	0.235		10 (13.89)	18 (25.00)	0.299	0.584
Low expression	44 (37.29)	23 (19.49)				13 (18.06)	31 (43.06)		

### 2.7 生存分析

#### 2.7.1 Ki67表达与Ⅰ期肺腺癌DFS、OS的关系

Ki67高表达组5年DFS明显低于Ki67低表达组（HR=6.265, 95%CI: 2.804-13.996, *P* < 0.001）（图 2A），同样Ki67高表达组OS也明显低于Ki67低表达组（HR=8.519, 95%CI: 2.789-25.940, *P* < 0.001）（图 2B），说明Ⅰ期肺腺癌患者术后5年DFS及OS与Ki67表达呈负相关。

**图 2:**
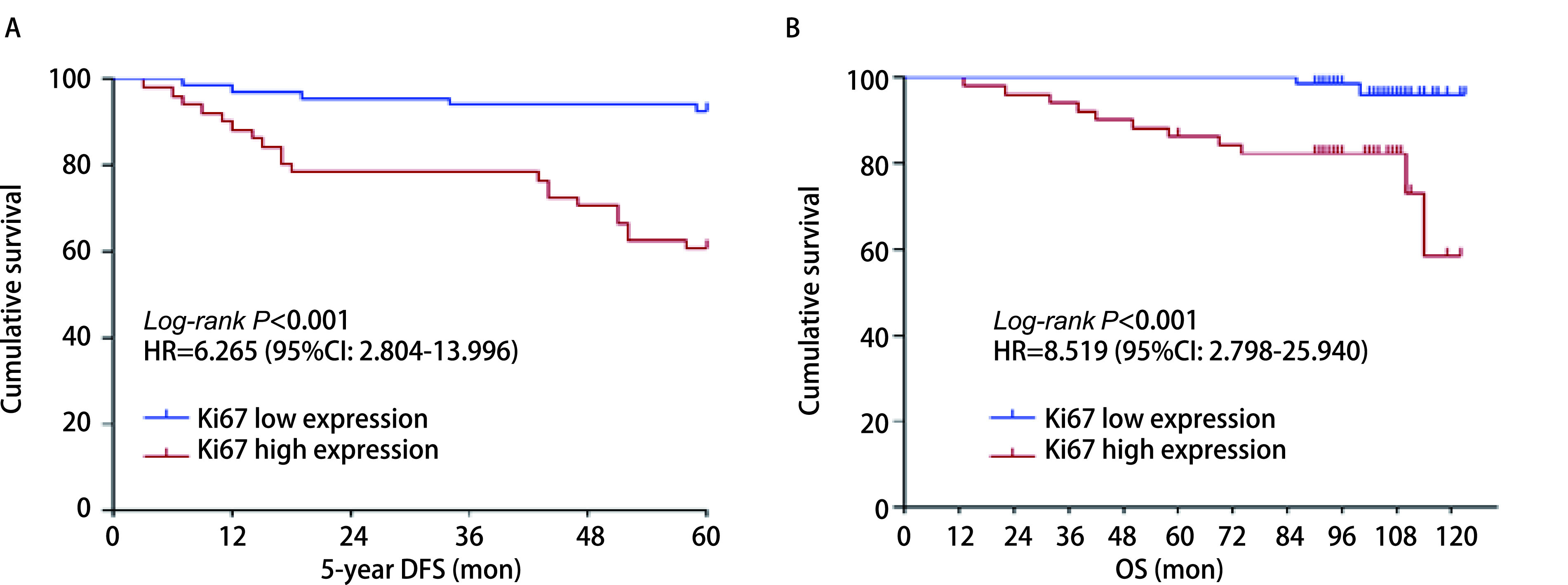
不同Ki67表达水平对Ⅰ期肺腺癌术后DFS、OS的影响。A：Ki67不同表达水平对术后DFS的影响；B：Ki67不同表达水平对术后OS的影响。 Effect of Ki67 expression on DFS and OS in patients with stage I lung adenocarcinoma. A: Effect of Ki67 expression on 5-year DFS in postoperative patients; B: Effect of Ki67 expression on OS in postoperative patients.

#### 2.7.2 *EGFR*基因突变与Ⅰ期肺腺癌DFS、OS的关系

*EGFR*突变型的5年DFS以及OS与EGFR野生型差异无统计学意义（*P* > 0.05）（图 3A和图 3B）。而在*EGFR*突变亚型中，*EGFR*外显子19缺失的5年DFS明显低于*EGFR*外显子21 L858R突变（HR=3.016, 95%CI: 0.968-9.394, *P*=0.031），差异有统计学意义（图 3C）；然而这两个亚组在OS上并无明显统计学差异（HR=0.350, 95%CI: 0.072-1.704, *P*=0.308）（图 3D）。

**图 3:**
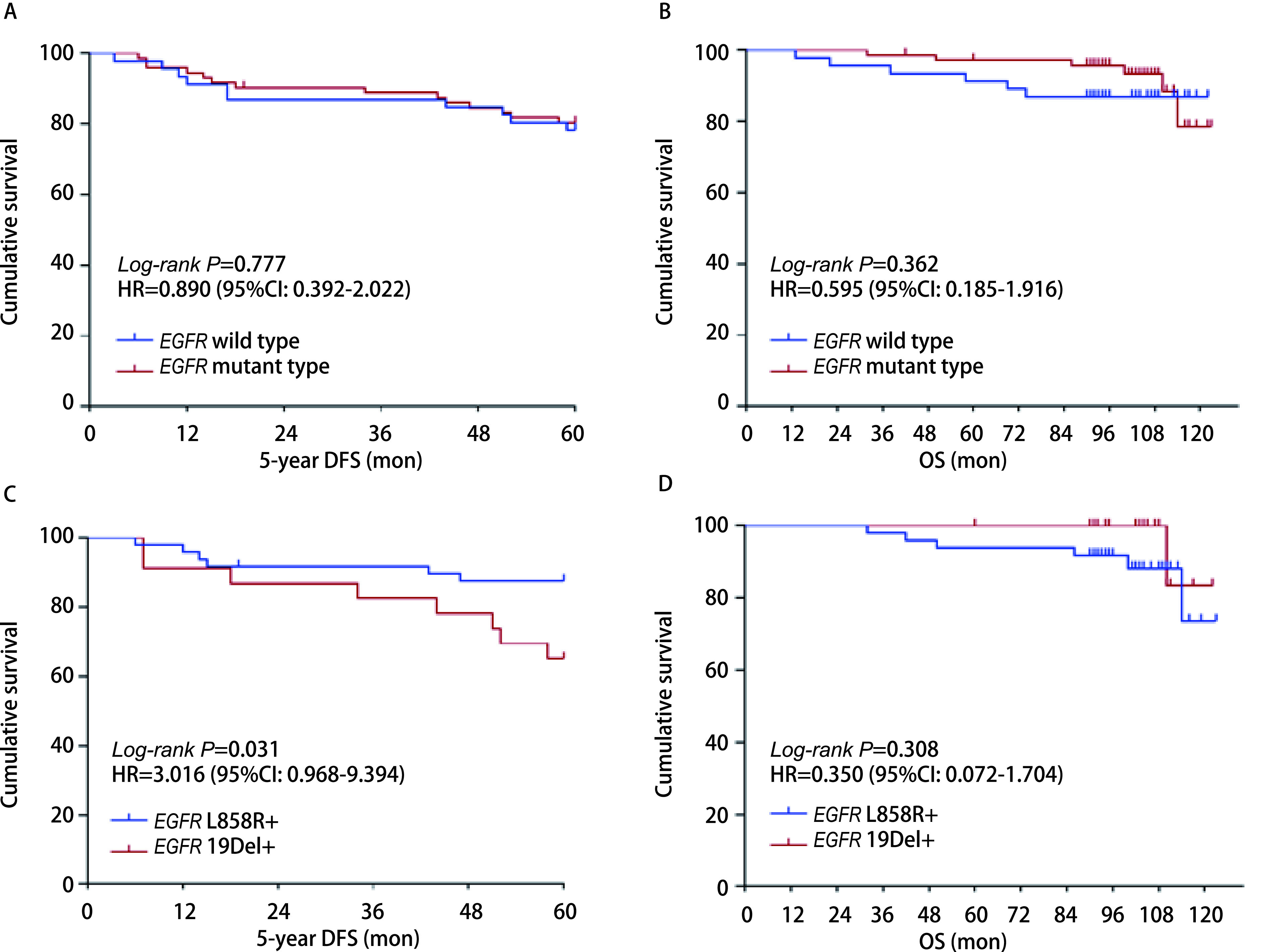
*EGFR*突变型及EGFR野生型对Ⅰ期肺腺癌术后DFS、OS的影响。A：*EGFR*突变型及EGFR野生型对术后DFS的影响；B：*EGFR*突变型及EGFR野生型对术后OS的影响；C：*EGFR*外显子19缺失及*EGFR*外显子21 L858R突变对术后DFS的影响；D：*EGFR*外显子19缺失及*EGFR*外显子21 L858R突变对术后OS的影响。 Effect of *EGFR* mutation status on DFS and OS in patients with stage I lung adenocarcinoma. A: Effect of *EGFR* mutation status on 5-year DFS in post-operation patients; B: Effect of *EGFR* mutation status on OS in post-operation patients; C: Effect of *EGFR* mutation type on 5-year DFS in post-operation patients; D: Effect of *EGFR* mutation type on OS in post-operation patients.

#### 2.7.3 一般临床特征与Ⅰ期肺腺癌DFS、OS的关系

118例Ⅰ期肺腺癌患者单因素分析显示，各项临床特征与Ⅰ期肺腺癌5年DFS均无显著相关性（*P* > 0.05）；而性别、肿瘤分化程度、肿瘤直径是Ⅰ期肺腺癌OS的预测因子（*P* < 0.05），年龄、是否吸烟、手术方式、术后分期、术后是否辅助治疗等无统计学差异（*P* > 0.05）（表 3）。

**表 3:** 临床特征与Ⅰ期肺腺癌DFS、OS的关系 Relation between DFS, OS and clinical characteristics in patients with stage I lung adenocarcinoma

Characteristics	5-year DFS				OS		
	HR	95%CI	*P*		HR	95%CI	*P*
Age (year)							
< 60	-	-	-		-	-	-
≥60	2.127	0.774-5.846	0.125		2.948	1.111-7.824	0.074
Gender							
Female	-	-	-		-	-	-
Male	1.937	0.861-4.364	0.103		8.029	2.655-24.280	0.007
Smoking status							
Never	-	-	-		-	-	-
Current or ever	1.314	0.545-3.170	0.543		2.022	0.661-6.186	0.208
Surgical method							
Lobectomy	-	-	-		-	-	-
Segmentectomy	3.278	0.771-13.943	0.088		3.227	0.419-24.853	0.184
Differentiation degree							
High	-	-	-		-	-	-
Medium and low	2.252	0.934-5.432	0.071		4.988	1.679-14.812	0.042
Stage			0.333				0.384
IA1	-	-	-		-	-	-
IA2	3.372	0.250-45.542	0.360		2.006	0.410-10.420	0.486
IA3	4.302	0.521-35.557	0.176		2.838	0.581-13.866	0.312
IB	4.117	0.600-28.263	0.150		4.053	1.062-15.473	0.150
Pleural invasion							
Yes	-	-	-		-	-	-
No	0.703	0.145-3.412	0.380		0.980	0.269-3.564	0.975
Tumor diameter (cm)			0.130				0.020
≤1	-	-	-		-	-	-
1-2	1.926	0.399-9.301	0.521		3.131	0.256-38.271	0.372
2-3	2.578	0.581-11.439	0.350		3.570	0.421-30.283	0.243
> 3	5.060	0.411-62.354	0.139		25.52	1.268-513.506	0.034
Postoperative treatment							
Yes	-	-	-		-	-	-
No	1.218	0.281-3.403	0.718		1.485	0.393-5.608	0.602

### 2.8 多因素分析

多因素分析结果显示：Ki67表达是影响Ⅰ期肺腺癌患者术后复发的独立预后因素（*P*=0.001），Ki67表达、性别是影响Ⅰ期肺腺癌患者术后总生存的独立预后因素（*P* < 0.05）（表 4）。

**表 4:** Ⅰ期肺腺癌患者术后复发及总生存的多因素*Cox*回归分析结果 regression analysis of postoperative recurrence and OS in patients with stage I lung adenocarcinoma

Characteristics	5-year DFS				OS		
	HR	95%CI	*P*		HR	95%CI	*P*
Ki67 (> 19.4%)	6.671	2.178-20.432	0.001		5.701	1.183-27.483	0.030
*EGFR* (Mutant type)	0.815	0.336-1.976	0.651		0.705	0.225-2.207	0.548
Gender (Male)	1.483	0.594-3.707	0.399		7.524	1.469-38.553	0.015
Differentiation degree (Medium and low)	1.147	0.434-3.033	0.782		1.681	0.334-8.470	0.529
Stage (IB)	1.377	0.585-3.240	0.464		1.904	0.595-6.093	0.278
Tumor diameter (> 2 cm)	1.361	0.568-3.263	0.490		2.156	0.660-7.037	0.203

## 3 讨论

目前，对于Ⅰ期肺腺癌术后辅助治疗存在争议，尤其是IA期，寻找影响早期肺腺癌患者术后复发及生存相关因素具有重要研究意义。本研究中我们整理了Ⅰ期肺腺癌术后复发及长期生存的相关数据，探讨Ki67的表达、EGFR突变状态及一般临床资料与预后的关系。

多项荟萃分析显示，Ki67高表达与肺癌不良预后相关。Wei等^[12]^发现Ki67高表达在不同肺癌病理亚型有更差的DFS和OS，但未行肺癌早期和晚期亚组分析。另一项涉及1, 931例Ⅰ期NSCLC患者的荟萃分析^[13]^，进行了亚组分析证实了Ki67对Ⅰ期肺腺癌的预后价值，且辅助治疗可能有益于Ki67高表达的Ⅰ期NSCLC患者。本研究显示Ki67的表达量在Ⅰ期肺腺癌中随着肿瘤分化程度越低、术后病理分期越高而升高，且Ki67高表达与男性、吸烟者显著相关（*P* < 0.05），与既往研究^[12]^一致。生存分析发现Ki67表达水平与早期肺腺癌DFS、OS呈负相关，且相较于Ki67低表达组，Ki67高表达组5年的DFS及OS的HR > 1，表明Ki67高表达组患者更易复发、生存率更低。此外，我们还进行了多因素分析，Ki67高表达组5年DFS的HR=6.671（95%CI: 2.178-20.432, *P*=0.001），OS的HR=5.701（95%CI: 1.183-27.483, *P*=0.03），更表明Ki67是Ⅰ期肺腺癌的独立预后标志物。

尽管很多研究表明Ki67与NSCLC预后密切联系，但目前在NSCLC中尚无临床相关分层的最佳且广泛适用的Ki67 Cut-off值，Lei等^[14]^研究得出Ki67 > 30%可作为与Ⅰ期-IV期NSCLC预后的预测因子，Warth等^[15]^研究表明Ki67 > 25%与Ⅰ期-IV期肺腺癌患者的不良预后相关，而Yu等^[16]^发现Ki67 > 13%是Ⅰ期肺腺癌的独立不良预后标志物。大量研究提供了不同的Ki67 Cut-off值，缺乏统一的度量阻碍了Ki67在NSCLC常规诊断中的使用，使得Ki67预后分级缺乏特异性，造成这一现象的可能因素有：①大多数研究样本量小，且为单中心研究；②研究中混杂因素较多，不同亚型、不同分期的NSCLC，不同病理类型及不同病理分级的预后本身就有差异；③很多研究随机选定Ki67 Cut-off值；④Ki67通过免疫组化进行评估，观察染色组织的阳性细胞在细胞总数中的百分比，主观性不可避免。在本研究中，我们对Ⅰ期肺腺癌进行了统计分析，排除了淋巴结状态、远处转移或肿瘤类型的影响，Ki67的评估由两名高年资病理科医生进行，以尽量减少主观性的影响，最终通过ROC曲线分析得出Ki67（5年DFS）及Ki67（OS）的Cut-off值分别为19.4%、20.0%，数值相近，但本研究样本量较小，需扩大样本量进一步估算。

在NSCLC中，*EGFR*突变发生在酪氨酸激酶区域的外显子18-21，最常见的为外显子19缺失及外显子21错义突变（尤其L858R位点），分别占突变的45%及40%-45%，其余10%的突变涉及外显子18和20^[10]^。2009年发表于*New Engl J Med*上的IPASS研究^[11]^，显示肺癌在亚裔、女性、不吸烟、腺癌人群中发生*EGFR*突变较其他人群高，*EGFR*突变率达60%。很多研究证实了这点^[17, 18]^。而本研究中显示女性、不吸烟患者在早期肺腺癌中*EGFR*突变率高，总检出突变率达61.02%，与之相一致。Suda等^[19]^的一项关于5, 780例早期非鳞状NSCLC人群的回顾性分析发现与EGFR野生型患者相比，EGFR突变患者的无复发生存期（recurrence-free survival, RFS）和OS显著延长。而Ito等^[20]^一项多中心回顾性分析发现1, 155例pN0-1M0肺腺癌病例中，EGFR突变病例在IA1期-IB期/高危型中表现出更差的RFS。本研究中，生存分析显示*EGFR*突变状态与Ⅰ期肺腺癌患者术后DFS、OS无显著相关性，这与之前的其他研究^[21, 22]^一致。可见EGFR突变状态在早期肺癌术后患者中的预后影响存在明显争议，造成这种截然不同结论的可能原因有：①*EGFR*常见突变类型之间及罕见突变类型（如外显子18 G719X突变、外显子20插入突变、外显子20 S768I突变、外显子21 L861Q突变等）预后不同，且部分为复合突变，有的携带一些共突变基因（如*TP53*、*NF1*、*RB1*、*PTEN*基因等），或者有其他驱动基因突变（如*ALK*、*ROS1*、*MET*、*RET*等），这些混杂因素均会影响研究结果；②可能需要对复发风险进行更细致的分层，术后复发风险应在有不同复发风险人群中讨论。由于本研究EGFR检测方法简单，只显示单个因素，未能多基因检测，且样本量少无法再分组，结论可能存在偏倚；且考虑Ki67为强混杂因素，我们也曾尝试根据Ki67的表达分组，但遗憾的是由于样本量少，未能得出*EGFR*不同突变与预后的统计学差异。而本研究亚组分析发现，但*EGFR*外显子19缺失组较*EGFR*外显子21 L858R突变组显示更差的5年DFS，提示更易复发，但在OS上二者却相似，考虑与*EGFR*外显子19缺失组对EGFR-TKIs有更好的应答有关^[23]^，这与Suda等^[19]^研究一致，由于病例少，可能存在一定的误差，需要扩大样本量验证。

尽管*EGFR*突变的NSCLC的临床特征已得到充分研究，然而，*EGFR*突变与Ki67相关性及其预后价值仍不清楚。许多研究^[24-26]^发现，Ki67低表达时*EGFR*突变率高；Li等^[24]^亚组分析发现NSCLC中分别比较*EGFR*外显子19或21突变与EGFR野生型间的Ki67表达量无显著差异。而本研究显示*EGFR*突变状态与Ki67表达之间无明显相关性，Ki67高表达时，*EGFR*突变率为54.90%（28/51），Ki67低表达时，*EGFR*突变率为65.67%（44/67），跟既往研究有相似的趋势，但*P*=0.235，无统计学差异，且不同*EGFR*突变类型与Ki67表达亦无相关性。未能得到阳性结果，考虑与样本量过少、未统一的Ki67 Cut-off值、单一的肿瘤分期等有关。

另外本研究单因素及多因素分析未能观察到各项临床特征与Ⅰ期肺腺癌复发的相关性。而发现男性、较差的肿瘤分化、较大的肿瘤直径与Ⅰ期肺腺癌较差的OS显著相关；多因素分析发现男性（*P*=0.015）仍然是Ⅰ期肺腺癌术后较差OS的独立危险因素，这与文献数据^[16, 27]^研究一致。

同时，本研究的不足在于：首先，本研究为单中心回顾性研究，体量较小，且早期病例复发人群比率本身少、总生存率高，生存数据有限，造成本研究中的样本量较少，一些结论需要更大的样本量来验证；同时，本研究中的病例由于手术时间较早，检测方法相对简单，检测的分子生物标志物种类单一且较经典、联合预测预后的因素少，目前第二代测序（next generation sequencing, NGS）可进行多基因检测，在建立预测模型方面还有更大的改进空间；另外，收集数据主要通过电话随访及住院或门诊资料追踪，对数据分析准确性可能存在影响。

综上所述，Ki67表达是Ⅰ期肺腺癌术后复发及OS的独立影响因素，与Ⅰ期肺腺癌*EGFR*突变无明显相关；*EGFR*突变状态与Ⅰ期肺腺癌预后关系未见显著差异，但在Ⅰ期肺腺癌中，*EGFR*外显子19缺失较*EGFR*外显子21 L858R突变术后复发风险高。本研究的探讨为早期肺腺癌除外单纯基于解剖特征的TNM分期模式，转向联合多种分子标志物等综合性分析来提高Ⅰ期肺腺癌患者预后的准确性提供了思路。未来临床上需更多的前瞻性研究来探索多基因突变、更多的分子标志物与早期肺腺癌的预后关系，筛选高危人群，为可能需要术后辅助治疗的这部分人群提供依据，延长其生存时间。
